# 21^st^ Century - Still No Standardization

**Published:** 2009-02

**Authors:** Urmila Palaria, DC Punera, AK Sinha, UK Bhadani, B Chhabra

**Affiliations:** 1 Assistant Prof., Department of Anaesthesiology, UFHT Medical College, Haldwani- Nainital(uttarakhand); 2 Assistant Prof., Department of Anaesthesiology, UFHT Medical College, Haldwani- Nainital(uttarakhand); 3 Assistant Prof., Department of Anaesthesiology, UFHT Medical College, Haldwani- Nainital(uttarakhand); 4 Professor, Department of Anaesthesiology, UFHT Medical College, Haldwani- Nainital(uttarakhand); 5 Professor, Department of Anaesthesiology, UFHT Medical College, Haldwani- Nainital(uttarakhand)

**Keywords:** Standardization, Anaesthetic equipment, Endotracheal tube, Indian Society of Anaesthesiologists

## Abstract

**Summary:**

Standardization of anaesthetic equipment is needed for safe anaesthetic practice. Various organizations and regulatory bodies have been made throughout world to formulate and control standards for anaesthesia equipment including endotracheal tubes. All endotracheal tubes must conform to ASTM standards. This has medico-legal importance also. Regulatory bodies should look after the whole process right from the manufacturers to the actual users. The Indian Society of Anaesthesiologists promotes safe anaesthetic practice, by establishing purchase guidelines for equipments and drugs. It is working in collaboration with World Federation of Societies of Anaesthesiologists. Standards have made anaesthesia and critical care equipment much safer over the years. There is need to form standards for various equipment in India.

## Introduction

Likeany equipment, anaesthesia equipment should follow “Standards” in manufacture and use. All over the world Standard protocol is followed for manufacture and use of various anaesthesia equipments. This is required for safety of anaesthesia practice. This is necessary in present Indian scenario considering increasing consumer forum cases against anaesthesiologists. In this article various aspects of standardization process is discussed in brief. Endotracheal tubes are studied as example to think over the matter of standardization. There is need of standardization of endotracheal tubes, which is one of most commonly used equipment used by anaesthesiologists. Various brands of endotracheal tubes studied in this article is not an end in itself but taken as an example. There is not any intention, for proving any particular brand superior or inferior. The article is not intended to judge quality of any particular brand of endotracheal tubes mentioned and studied in the article. Its quality has to be judged by individual anaesthesiologist and regulatory bodies. The purpose of this article is to draw attention of anaesthesiologists in India to bring the role of standardization into thought process, in respect of anaesthesia equipment. Role of Indian Society of Anaesthesiologists is also thought for as a regulatory body for safe anaesthesia practices. It is twenty first century and still there is no standardization.

## What is ‘Standard’?

Standard can be defined as “Documented agreements containing technical or performance specifications or other precise criteria to be used consistently as rules, guidelines or definitions of characteristics, to ensure that materials, products, process and services are fit for their intended purposes”^1^.

Three types of standards in Anaesthesia and Critical care:

**Safety standards:** Minimum requirements for electrical safety and usability.

**Performance safety standard:** Minimum requirement for equipment performance during use.

**Technical standard:** Provide guidance to manufacturers and users for equipment design, construction, performance and use.

## How does process of Standardization starts?

Process of standardization usually starts when a request from manufacturer, industry association, consumer group, educational institute or governmental body is made, to help with a particular safety, performance or quality issue. Initial drafts are written by working groups or adapted from other countries or from standards of similar equipment. National committee members are asked to comment and vote on it. Standards are developed by technical committees, subcommittees and working groups made up of representatives of manufacturers, equipment users, operators and other interested parties using a consensus approach.

## Standards for Anaesthesia and Critical Care

Most of anaesthesia and critical care standards are written by ISO TC 121 and its subcommittees, Organisation for International Standardisation Technical Committee 121 and IEC 62-International Electro technical Commission Committee 62. These committees look for suggestions of anaesthesiologists and intensivists, apart from engineers.

## What are ISO and IEC?

**ISO** stands for International Organisation for Standardisation, Founded in Feb 1947, first met in London UK in 1967^2^ and present headquarters in Geneva, Switzerland^3^. National standards bodies of over 140 countries formed this federation. This body has developed a series of ‘standards’ applicable to various aspects of economic activity – manufacturing as well as services. Institutes that demonstrate compliance with these Standards are certified by ISO with respect to that Standard. Some examples for different certification used for different purpose are, Certification for quality- ISO9000 (QMS) series Certification for environment- ISO14000 (EMS) series, certification for occupational safety-OHSAS 18000(OHSMS) series etc.

**IEC,** International Electrotechnical Commission, originally located in London, the commission moved to its current headquarters in Geneva in 1948^4^. It has 42 member countries. Members are drawn from principal standardization bodies from different nations. The IEC maintains advisory committees on electrical, medical and telecommunications, electronics on electromagnetic compatibility and safety.

## ‘Standard's’ bodies and organizations across the world

There are numerous standards writing committees and organizations across the world. Most countries have a national standards body:

**In United States:** Anesthetic and Critical Care Committee F29 of American Society for Testing and Materials (ASTM) writes the standards. ASTM has a dominant role among standards developers in the USA and claims to be the world's largest developer of standards.

**In Canada:** CSA international (formerly the Canadian Standards Association), is a global leader in development and certification of equipment standards, International Organisation for Standardisation (ISO), the International Electrotechnical Commission (IEC) and the Compressed Gas Association (CGA).

**In Europe:** Committee for European Normalization (CEN) and their marking is CE.

**In Japan:** Japanese Industrial Standards Committee (JISC).

**Standards in India:** Bureau of Indian Standards (BIS) is a member of “International Organisation for Standardisation (ISO)”.

### Bureau of Indian Standards (BIS)/Indian Standards

This is national standard's body in India; head-quarters is in New Delhi, a member of ISO. It has a training centre is in Noida (U.P.) known as National Institute of Training for Standardisation (NITS). NationalCommittees may decide to change or modify any of International Standards adopted for its own country. Its objectives are^5^………

-Harmonious development of standardization, marking and quality certification.

-Providing new thrust to standardization and quality control.

-Evolving national strategy for standards and integrating them with growth & development of production & exports.

BIS is engaged in formulation of Indian Standards for medical equipment and hospital planning. The Indian Standard is technically equivalent to ISO/IEC standard.

## Why standardization is needed in anaesthesia?

To reduce the discripencies among various manufacturers of Endotracheal tubes (ETTS), the need for minimum standards for safety in the design and construction of medical equipment were recognized across the world.

It is medico legally important for physician that he has to act in accordance with specific standards of care established by the profession for protection of the patient against unreasonable risks^6^. Jeffrey Cooper in his classic article in the journal Quality and Safety in Health Care, reprinted in 2002, pointed out that 84% of preventable incidents were due to human error and 14% of critical incidents in anaesthesia were related to equipment failure^7^.

Safety in anaesthesia practice relates safety in various aspects like use of drugs, procedures and equipments. It involves its make and knowledge of use. Any compromise or deviation from specific standard at any level may introduce risk to patient safety. These deviations can creep in by oversights in design, mistakes in equipment manufacturer or facility construction and the need to produce a usable product for the price the health care system can afford, especially in developing countries. Standards provide similarity in equipment design and materials across different models and different manufacturers. The manufacturer owes his duty of care to the ultimate user and not just to the immediate purchaser. Until recently, anaesthesia and critical care equipment produced by single manufacturer had to adhere to standards of all the countries in which equipment was distributed. This led to the rise in actual price of equipments. Now global standards are being harmonized so that manufacturers in future will only have to manufacture equipment to one international standard^1^.

The Global Harmonization Task Force (GHTF) was conceived in 1992 in an effort to respond to growing need for international harmonization in the regulation of medical devices.

By standardization, what we are really trying to do is to write the minimum requirements for the “basic safety and essential performance” for medical equipment in general or for a particular device^1^.

**Endotracheal tubes (ETT)** are one of most commonly used equipment used by anaesthesiologists throughout the world. We will like to discuss issues related with endotracheal tube's standards in Indian perspective.

**Endotracheal Tubes marketed in India: Does it follow standards?**

What are different standards for Endotracheal tubes?

**ASTM** standard requires following recommendations to be done and printed on endotracheal tubes^8^:

- Name or trademark of manufacture or supplier

- The words “oral”, “nasal” or “oral/nasal”

- Inside (ID) and outside (OD) diameters in millimetres

- Tissue toxicity test: Implantation testing (IT) or other, with Notation Z-79 or F-29 (as per testing),

- Length (depth) markings in centimetres measured from patient end.

- Cautionary note such as ‘Do not reuse’ or ‘Single use only’, if disposable

- Radiopaque marker at patient end or along the full length.

## Comparison of ETT of Different Manufacturer marketed in India

We collected some of the common brands of Endotracheal tubes available in Indian market and compared them according to ASTM standards. We chose to study one of the most commonly used tube size of 7.5 (Table: 1)

**Table 1:** Comparison of ETT of Different Manufacturer marketed in India

Manufacturer/supplier			Data printed on the endotracheal tubes				
printed on cover paper	Name of manufacturer printed on tube	Size (ID)	O.D.	Total Length	Starting point of marking	Black bars	Cuff design
Portex	Yes	7.5mm	10.3mm	28	18cm	Single	Oval with lesser contact surface area
Romson	Yes	7.5mm	10mm	28	18cm	Double	More ridges with larger Contact surface area
Polymedicore	No	7.5mm	10mm	26	18cm	-	More ridges with larger Contact surface area
Umiya Surgical	No	7.5mm	10mm	27	19cm	-	More ridges with larger Contact surface area
Zhanjiang star	No	7.5mm	10mm	30	20cm	Double thin	Oval with lesser contact surface area

**Fig 1:**
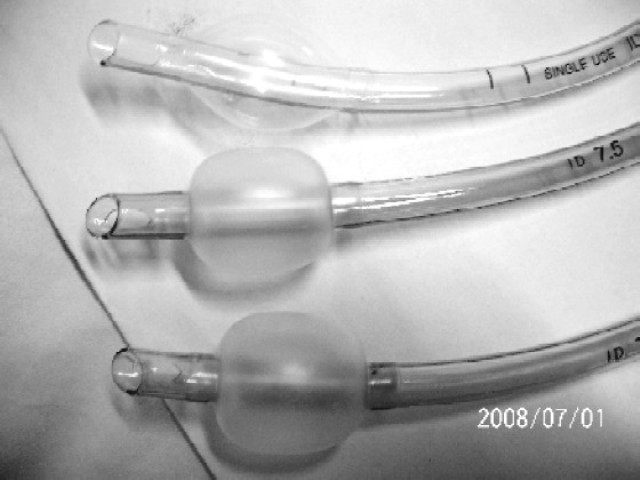
Endotracheal tubes showing different cuff shapes without any manufacturer name

**Fig 2:**
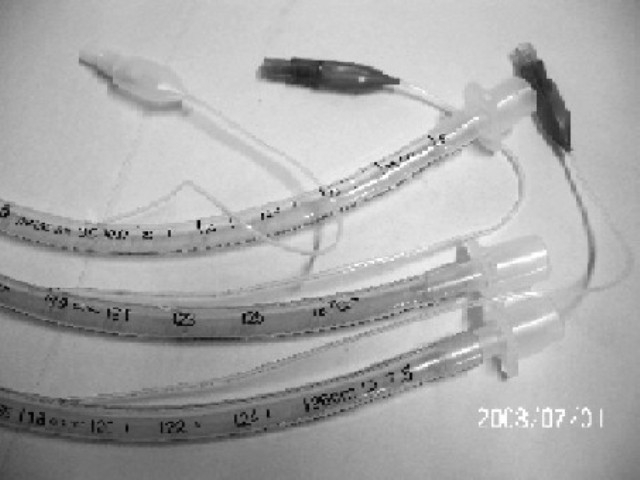
Endotracheal tubes showing different take off point of pilot balloon tube, different length of tube and outer diameter of 10.0

**Fig 3:**
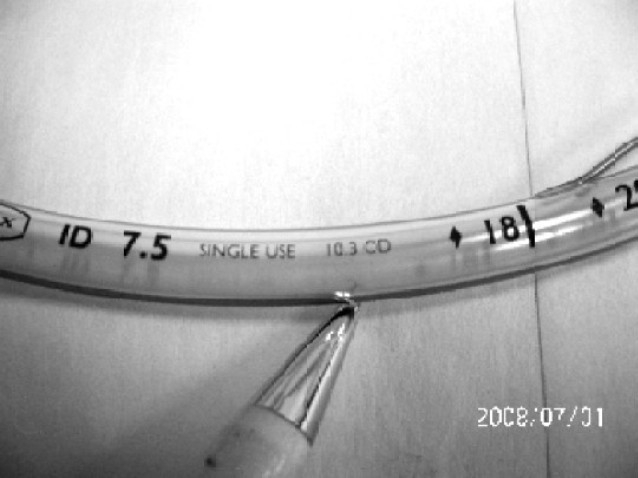
Endotracheal tube showing Outer Diameter (OD)- 10.3 (c.f. other tubes having OD 10.0)

Endotracheal tubes studied from different manufacturer were compared and following findings were noted:

1. Some of tubes have no markings related with manufacturer / supplier on the tube. Once tube taken out of packages, one can't know which Manufacturer /supplier it belongs to (Fig 1).
2. Many of ETTs have no indication of toxicity test on the tubes.
3. Tubes of same size vary in their length markings at machine end i.e. 26, 27, and 30 cm (Fig 2). Take off point of pilot balloon is different in different tubes. ASTM does not comment anything on this aspect.
4. In spite of considering same internal diameter tubes of 7.5mm, most of the tubes have outer diameter of 10 but one tube has outer diameter of 10.3mm (Fig 3).
5. Many tubes have ring marking to help position the tube in relation to entry point into trachea i.e. vocal cords^9^. This becomes more important when EtCO_2_ monitoring is not available to confirm correct positioning of tube.
6. ASTM does not comment anything about cuff design (contour) but it becomes important as it changes the contact surface area on trachea and may be of importance. In our study some of the cuffs were oval and some attend circular when inflated outside as shown in Fig 1. Its pressure and shape inside trachea in vivo is a matter of further study.
7. Place of Murphy Eye is also not fixed in relation to end of ETT. ASTM does not comment on this.

## What should be done to keep up to Standards?

As an expert of the subject of Anaesthesiology it is our duty to keep ourselves updated for different equipments we use. We should know about various international standards of that equipment. As an end user it is our duty to keep the safety of patient at the top priority. Equipment safety is one of important constituent of safety in anesthesia. As a purchaser and policy maker this becomes more important because it deals with multiple users and community as a whole.

## Role of Regulatory bodies

**Various governmental and non-governmental bodies** are responsible for making of laws and regulations for the manufacture, use, and maintenance of various medical equipments. However, anaesthesiologists need to encourage their governmental regulatory bodies to encourage manufacturer compliance with specific standards in order to increase patient safety. Other than anaesthesiologists every hospital and organizations should have regulations for purchase and maintenance of various equipment of medical use. Role of Indian Society of Anaesthesiologists becomes important in case of Speciality of Anaesthesia. It is the National anaesthesia society and it promotes safe anaesthetic practice by establishing purchase guidelines for Anaesthesia equipments and drugs^9^. The Indian Society of Anaesthesiologists should discuss the several aspects of legal and other matters to standardize medical equipments. Society can be instrumental in raising awareness about understanding standardization by its role as a forum for improvement in anaesthesia practice in India. Quality, excellence and cost-effectiveness are the interrelated factors and must be part of everyday practice in anaesthesia^10^.

We have taken endotracheal tubes as an example of one of most commonly used simple equipment by anaesthesiologists. Similarly other equipments like various monitors, anaesthesia machines, pipeline etc can be studied in Indian and International scenarios to improve upon our practice of anaesthesia to achieve the goal of safe anaesthesia practice.
